# Maladie d’Ebstein révélée par une anasarque fœtoplacentaire: à propos d’une observation originale

**DOI:** 10.11604/pamj.2016.24.279.9970

**Published:** 2016-07-28

**Authors:** Hanaa El Hadraoui, Amina Barkat

**Affiliations:** 1 Equipe de Recherche en Santé et Nutrition du Couple Mère Enfant, Service de Médecine et de Réanimation Néonatale à l’Hôpital d’Enfant de Rabat, Faculté de Médecine et de Pharmacie, Université Mohammed V, Rabat, Maroc

**Keywords:** Anasarque, fœtoplacentaire, Ebstein, réanimation, néonatale, Hydrops, fetalis, Ebstein, reanimation, neonatal

## Abstract

La maladie d'Ebstein est une malformation cardiaque congénitale rarement révélée par une anasarque fœtoplacentaire. Nous rapportons une observation originale de maladie d’Ebstein, diagnostiquée au cours du bilan étiologique d’une anasarque, révélée par une échographie anténatale, objectivant un hydramnios, une ascite et un épanchement péricardique. L’échographie cardiaque a permis la mise en évidence d’une maladie d’Ebstein avec insuffisance tricuspide importante, une insuffisance mitrale grade 3 et un canal artériel perméable. La fermeture du canal artériel persistant, associée à la baisse des résistances pulmonaires par une ventilation optimale, a permis une amélioration hémodynamique et la survie du patient.

## Abstract (en)

Ebstein’s anomaly is a congenital heart defect rarely revealed by fetal-placental anasarca. Our study reports an original case of Ebstein’s anomaly diagnosed during fetal-placental anasarca assessment, revealed by antenatal ultrasound, objectifying hydramnios, ascites and pericardial effusion. Echocardiography allowed the identification of Ebstein’s disease with significant tricuspid insufficiency, mitral regurgitation (grade 3) and patent ductus arteriosus. The closure of the ductus arteriosus associated with the decrease of pulmonary resistance using optimal ventilation allowed hemodynamic improvement and patient survival.

## Introduction

La maladie d'Ebstein est une malformation cardiaque congénitale complexe rare de la valve tricuspide, décrite la première fois par Wilhelm Ebstein en 1866 [1]. Elle intéresse une naissance sur 200 000 et représente moins de 1% des malformations cardiaques congénitales [2, 3]. Nous rapportons une observation originale de maladie d’Ebstein révélée en anténatal par une anasarque fœtoplacentaire avec une bonne évolution.

## Patient et observation

Nouveau de sexe féminin, à terme, eutrophe, issu d’une grossesse mal suivie et d’un mariage consanguin de premier degré. La mère est âgée de 40ans, 9^ème^ geste 6^ème^ pare (3 avortements précoces de causes non documentées), groupage A+, Anamnèse infectieuse faite de leucorrhées et de brûlures mictionnelles non traitées. Une échographie faite la veille de l’accouchement a objectivé un hydramnios et des signes d’anasarque: une ascite et un épanchement péricardique. L’accouchement s’est effectué par voie basse, le nouveau-né a présenté une mauvaise adaptation à la vie extra-utérine; le score d’Apgar était à 3 à la première minute et à 8 à la cinquième minute après réanimation. Il avait un poids de 3100g.

À l’admission, le nouveau-né avait une détresse respiratoire à 4/10 selon le score de Silverman, une fréquence respiratoire à 70 cycles/min, la présence d’un souffle cardiaque et d’une hépatomégalie avec une tension artérielle à 87/47; La conduite à tenir initiale a consisté en une intubation et ventilation, la mise en place d’un cathéter veineux ombilicale, une ration de base avec des électrolytes et la mise sous antibiothérapie.

Au bilan biologique réalisé; le gaz du sang a révélé un pH à 7.02 et une PCO2 à 58. A la numération formule sanguine l’hémoglobine était à 15.9, les globules blancs à 248000 et les plaquettes à 121000. La CRP était négative. La fonction rénale était correcte avec une urée à 0.38 et une créatinine à 6.8. La PCR CMV et Parvovirus B19 sont revenues négatives.

Le nouveau-né était de groupe A Rh- C-c+E-e+K-, la mère était de groupe A Rh+ C+c+E-e+K- avec un Combs négatif. Une radiographie pulmonaire a montré une cardiomégalie avec un index cardiothoracique à 0,76. L’échographie transthoracique a objectivé une maladie d’Ebstein avec insuffisance tricuspide importante, une insuffisance mitrale grade 3 et un canal artériel perméable. L’échographie transfontanellaire a montré une dilatation ventriculaire minime et l’échographie abdomino-rénale a objectivé une ascite de grande abondance. Le diagnostic retenu est une anasarque non immunologique sur cardiopathie congénitale: maladie d’Ebstein. Le patient a nécessité une intubation ventilation assistée de trois jours avec restriction hydrique, lasilix à 1mg/kg/8H et un support hémodynamique noradrénaline et dopamine. Il a était extubé à quatre jours de vie et a nécessité une oxygènodépendance de huit jours. A la sortie, le nouveau-né avait un poids à 2800g une taille à 50 cm et un périmètre crânien à 34 cm. Le dernier exam clinique a été effectué à l’âge de 9 mois, le nouveau-né était bien portant, il avait un poids à 7900g, une taille à 69cm et un périmètre crânien à 41cm.

## Discussion

L’anasarque fœtoplacentaire est une pathologie sévère correspondant à une perturbation majeure de l’équilibre osmotique et oncotique fœtoplacentaire. La définition de l'anasarque fœtale est échographique: il s'agit de l’association d’un œdème sous-cutané diffus, supérieur à 5 millimètres et un épanchement dans une séreuse (péricarde, plèvre ou péritoine). Ou la présence d'un épanchement dans deux séreuses au moins sans œdème sous-cutané. Il y a quelques années, la majorité des anasarques fœtales étaient considérées comme secondaire à une incompatibilité rhésus fœto-maternelle, on estime désormais au contraire que plus de 90% des cas d’anasarques sont non immunologiques [4].

Les étiologies cardiaques sont à l’origine de 10% à 20% des cas d’anasarques non immunologiques [5, 6]. Parmi celles-ci, on trouve les anomalies structurelles, les arythmies cardiaques et les tumeurs [7]. Les anomalies du retour veineux, les anomalies et obstruction ventriculaires entrainent une diminution du débit cardiaque, puis une insuffisance cardiaque et une augmentation de la pression dans la veine ombilicale.

La maladie d’Ebstein est rarement révélée par une anasarque, il s’agit d’une anomalie de la valve tricuspide dont les feuillets septal et postérieur sont, par défaut de délamination, accolés à la paroi ventriculaire et déplacés vers la pointe du ventricule droit [8]. Le ventricule droit est ainsi atrialisé et l’oreillette droite est dilatée. Ce déplacement proximal des feuillets rend la valve tricuspide plus ou moins fuyante. Le degré de déplacement et de l’insuffisance tricuspide est variable, et il s’ensuit des formes plus ou moins graves de cette anomalie [9].

Du point de vue embryologique, les mécanismes aboutissant aux malformations de la valvule tricuspide dans l’anomalie d’Ebstein ne sont pas encore complètement élucidés. Ils résultent d’un défaut ou d’une absence de délamination au niveau des valves postérieure et septale de la tricuspide. Les conséquences physiopathologiques de cette malformation sont une insuffisance tricuspide, une désynchronisation mécanique intra-atriale par activation séquentielle du segment atrial vrai et du segment ventriculaire atrialisé, l’existence d’un shunt auriculaire droite-gauche responsable d’une cyanose, une atrésie pulmonaire fonctionnelle dans les formes sévères d’Ebstein néonatale rendant la cardiopathie ducto-dépendante.

L'augmentation de la pression hydrostatique intravascu¬laire, consécutive à une insuffisance cardiaque peut être responsable d’une anasarque. L’histoire naturelle de l’anomalie d’Ebstein est difficile à appréhender de façon simple en raison du large spectre du tableau clinique qu’elle offre : découverte fortuite chez le fœtus lors d’une échographie per-partum, cyanose chez le nouveau-né, insuffisance cardiaque chez le nourrisson, souffle cardiaque chez l’enfant et troubles du rythme chez l’adolescent et l’adulte [10].

La maladie d’Ebstein chez notre patient a été découverte lors du bilan étiologique d’une anasarque fœtoplacentaire de découverte anténatale. Le diagnostic se fait par échographie cardiaque avec mise en évidence d’un déplacement apical des feuillets tricuspides, une insuffisance tricuspide souvent majeure et une oreillette droite dilatée.

La sévérité de l’atteinte est décrite dans la classification de Carpentier [11]. Type I (moins grave): la valve tricuspide antérieure est plus grande et mobile, mais la partie postérieure et septale sont déplacées apicalement, dysplasiques, ou absents. La taille de la chambre ventriculaire atrialisée varie de relativement petite à grande. Type II: les valves antérieure, postérieure, et septales sont présents, mais sont relativement petits et déplacés dans un mode de spirale vers l'apex. La chambre ventriculaire atrialisée est modérément grande. Type III: la valve antérieure a un mouvement limité avec des cordages courts et fusionnés. L'insertion directe des muscles papillaires sur la valve antérieure est souvent présente. Les valves postérieure et septale sont déplacées, dysplasique, et généralement pas reconstructible. La chambre ventriculaire atrialisée est grande. Type IV (la plus grave): La valve antérieure est fortement déformée et déplacée dans la voie d'éjection du ventricule droit. Il peut y avoir ou pas quelques cordages, et les insertions directes des muscles papillaires dans le bord de la valve sont communs. La valve postérieure est typiquement dysplasique ou absente, et la valve septale est représentée par une nervure de matériau fibreux à partir de l'apex du septum membraneux. Le tissu de la valve tricuspide est déplacé et peut causer l'obstruction de la circulation sanguine (sténose tricuspide fonctionnelle). Presque toute la cavité ventriculaire droite est atrialisée.

Notre patient a une maladie d’Ebstein avec insuffisance tricuspide importante, une insuffisance mitrale grade 3 et un canal artériel perméable. La prise en charge varie selon la sévérité de l’atteinte cardiaque. La maladie d’Ebstein est une cardiopathie ducto-dépendante, le traitement par les prostaglandines n’a pas été proposé à notre patient, il a bénéficié d’une ventilation, de support hémodynamique noradrénaline et dobutamine, associé au furosémide.

En effet, l’équipe de Bruckeimer a rapporté le cas d’un nouveau-né ayant une maladie d’Ebstein de diagnostic prénatal, chez qui le traitement par prostaglandine E1 a été interrompu devant l’instabilité hémodynamique et le shunt ductal gauche-droit alimentant la fuite pulmonaire. La fermeture du canal artériel a permis une amélioration hémodynamique et respiratoire mais aussi la diminution des résistances pulmonaires, permettant une diminution de la post-charge à laquelle est soumis le ventricule droit pathologique et l’amélioration de son débit, par une ventilation optimale et un traitement par NO inhalé lors d’un shunt circulaire [9, 12].

L’indication de plastie de la valve tricuspide reste très discutée. Les méthodes chirurgicales sont variées (réparation univentriculaire ou biventriculaire). Le pronostic est généralement sombre et la présentation clinique bruyante lorsque la pathologie se manifeste au cours de la vie fœtale ou lors des premiers mois de vie (anasarque, mort intra-utérine, décompensation cardiaque avec cyanose et parfois tableau d’atrésie pulmonaire fonctionnelle). Le taux de survie chez le fœtus avec une maladie d’Ebstein ou une dysplasie de la valve tricuspide est de 39 % à la naissance et d’environ 20 % à 1 mois de vie [13].

## Conclusion

L’observation que nous rapportons est d’un nouveau-né ayant une maladie d’Ebstein révélée par une anasarque fœtoplacentaire diagnostiquée sur une échographie anténatale. L’échographie cardiaque a objectivé une maladie d’Ebstein avec insuffisance tricuspide importante, une insuffisance mitrale grade 3 et un canal artériel perméable. La fermeture du canal artériel persistant, associée à la baisse des résistances pulmonaires, a permis une amélioration hémodynamique et une bonne évolution.
